# Editorial Note: Cutaneous Squamous Cell Carcinoma (SCC) and the DNA Damage Response: pATM Expression Patterns in Pre-Malignant and Malignant Keratinocyte Skin Lesions

**DOI:** 10.1371/journal.pone.0303120

**Published:** 2024-05-02

**Authors:** The PLOS ONE Editors

Following the publication of this article [1], concerns were raised regarding results presented in Figures 3, 7, 8, 9, and 10. Specifically,

- In Figure 3, the UV 1hr results and the UV 2hr results appear similar
- The following panels appear to partially overlap:
  - Figure 7A panel 2, Figure 7B panel 4, and Figure 10A bottom panel
  - Figure 7A panel 3, and Figures 7B panel 3 and 9B bottom panel when rotated
  - Figure 7A panel 4 and Figure 8B bottom panel
  - Figure 7A panel 5, Figure 7B panel 5, and Figure 8A bottom panel
  - Figure 7B panel 2 and Figure 9B bottom panel

Regarding the similarities within the Figure 3 panels, the corresponding author stated that an error was made when preparing the UV 1hr and UV 2hr panels, but they were unable to say which is correct in the published figure. Due to the time since the experiment was conducted, the original data underlying the Figure 3 results are no longer available. In the absence of the original data underlying Figure 3 this issue cannot be fully resolved. However, the corresponding author stated that the results in Figures 1, 2, and 6 support the findings reported for Figure 3, that pATM is transiently detected in the nucleus following UV irradiation. The Figure 1 and 2 versus Figure 6 results differ in whether the nuclear localization is still observed at the 2-hour timepoint, but all three figures support that pATM staining is largely cytoplasmic and perinuclear 4 hours after UV treatment.

Regarding the similarities between panels presented in Figure 7 and panels presented in Figures 8–10, the authors explained that Figure 7 intentionally uses panels from other figures to demonstrate the scoring of pATM staining intensity in the nucleus (7A) and cytoplasm (7B).

The authors provided image data files underlying the results presented in Figures 7, 9, and 10, as well as the original uncropped blots underlying the Figure S2 results.

The *PLOS ONE* Editors issue this Editorial Note to notify readers of the unresolved panel duplication in Figure 3 and to provide the above clarification about the intentionally duplicated panels presented in Figures 7–10.

## Supporting information

S1 File Original data underlying Figure 7. (ZIP)

S2 File Original data underlying Figure 9. (ZIP)

S3 File Original data underlying Figure 10. (ZIP)

S4 File Blot data underlying Figure S2. (ZIP)

## References

[1] Ismail F, Ikram M, Purdie K, Harwood C, Leigh I, Storey A (2011) Cutaneous Squamous Cell Carcinoma (SCC) and the DNA Damage Response: pATM Expression Patterns in Pre-Malignant and Malignant Keratinocyte Skin Lesions. PLoS ONE 6(7): e21271. doi: 10.1371/journal.pone.0021271 21747934 PMC3128585

