# Supplementary figures and images for: Editorial Note: Cutaneous Squamous Cell Carcinoma (SCC) and the DNA Damage Response: pATM Expression Patterns in Pre-Malignant and Malignant Keratinocyte Skin Lesions
Source: PLoS One. 2024 May 2;19(5):e0303120. doi: 10.1371/journal.pone.0303120 (PMC11065302; doi:10.1371/journal.pone.0303120)

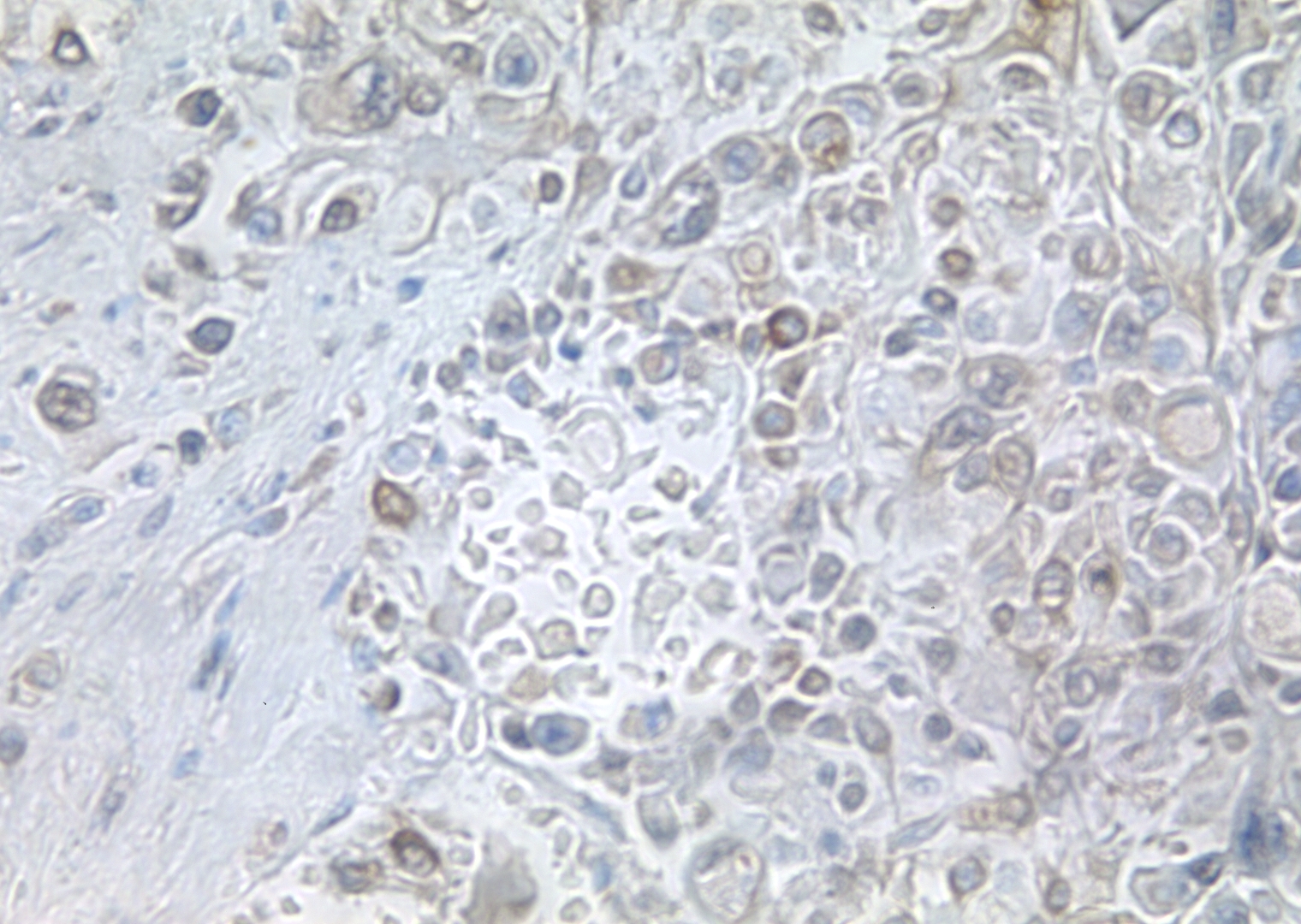

Supplement: S1 File — (ZIP) [file pone.0303120.s001.zip › Fig 7/Fig 7A 1.jpg]

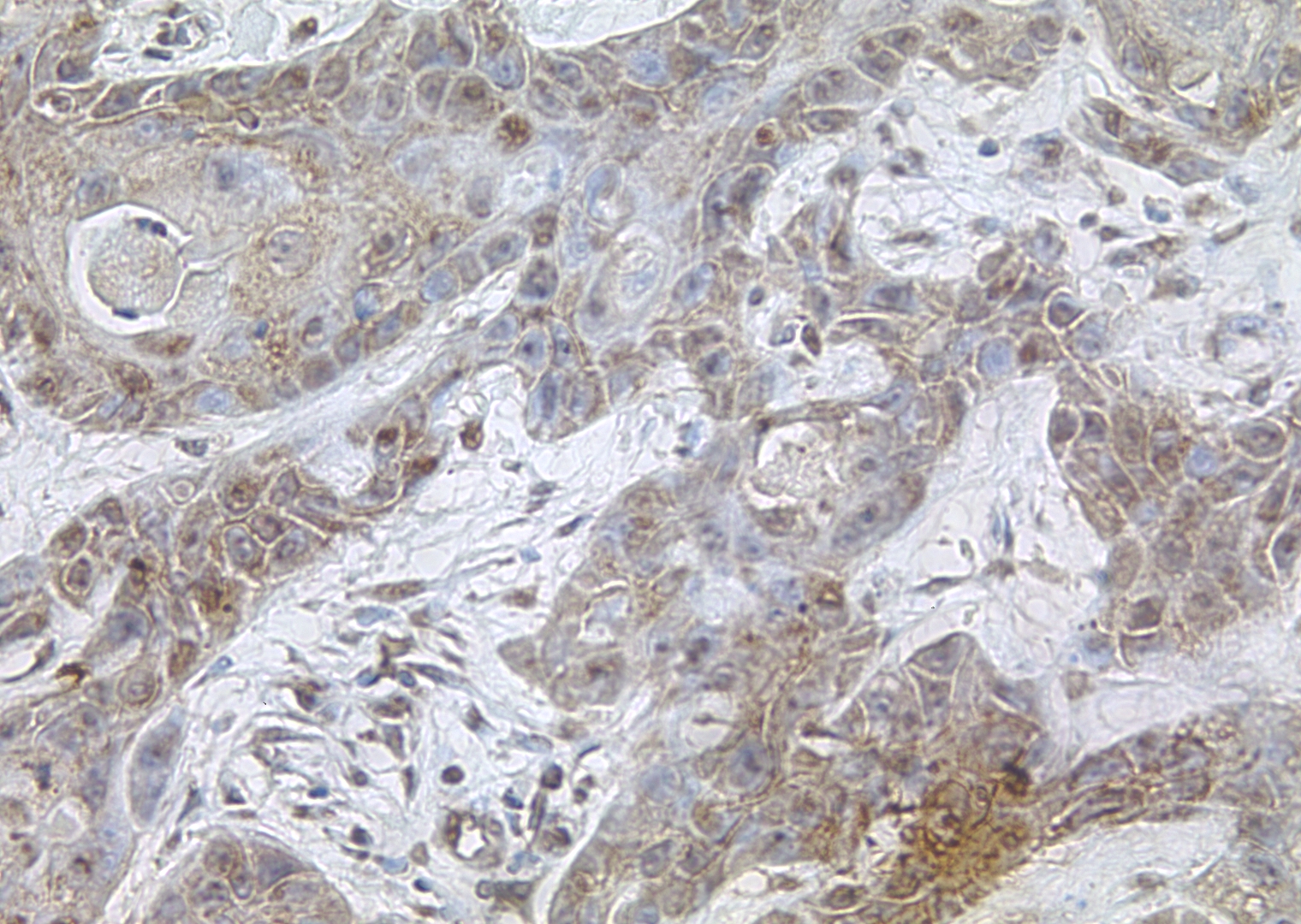

Supplement: S1 File — (ZIP) [file pone.0303120.s001.zip › Fig 7/Fig 7A 2 and Fig 7B 4.jpg]

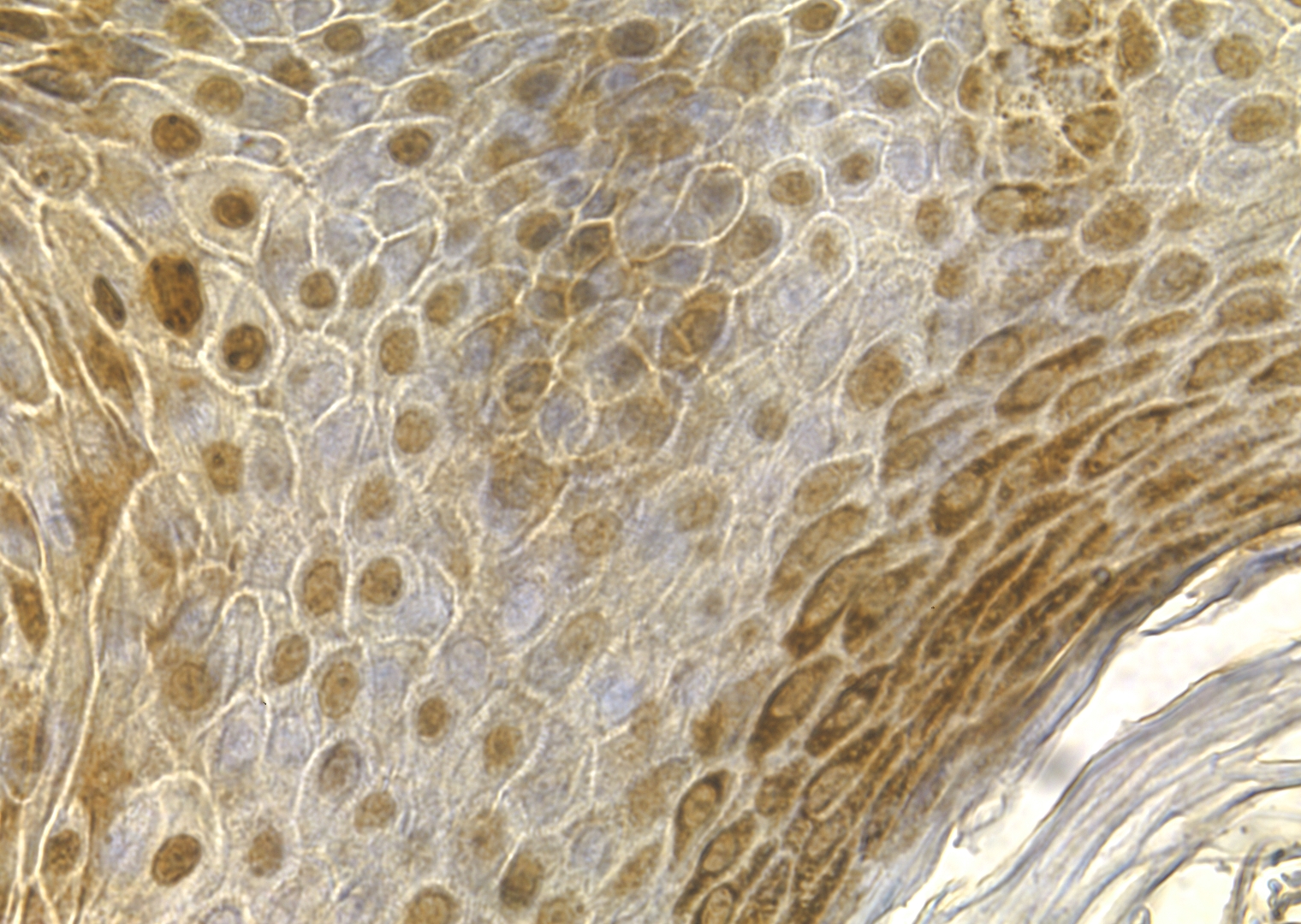

Supplement: S1 File — (ZIP) [file pone.0303120.s001.zip › Fig 7/Fig 7A 3 and Fig 7B 3.jpg]

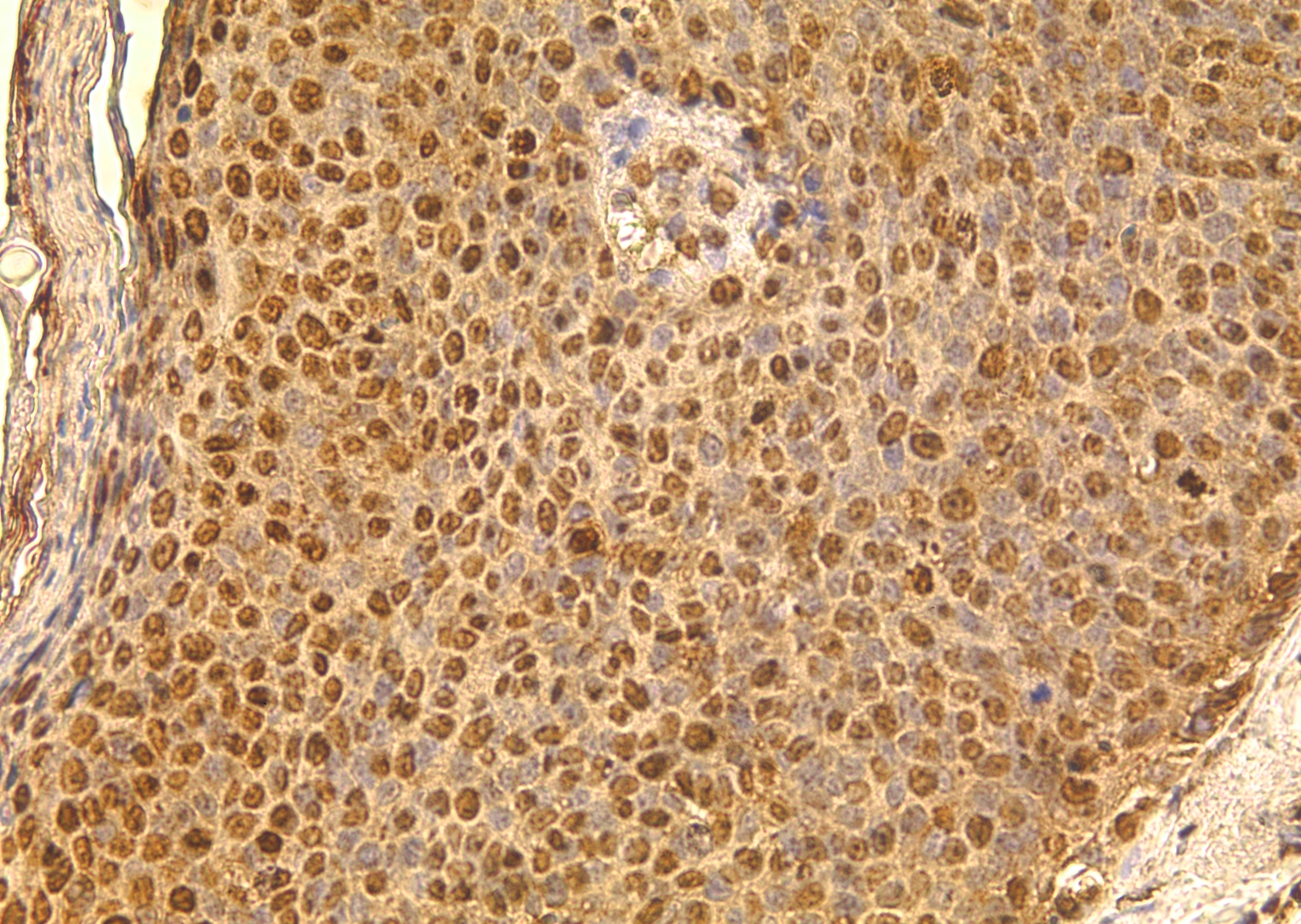

Supplement: S1 File — (ZIP) [file pone.0303120.s001.zip › Fig 7/Fig 7A 4.jpg]

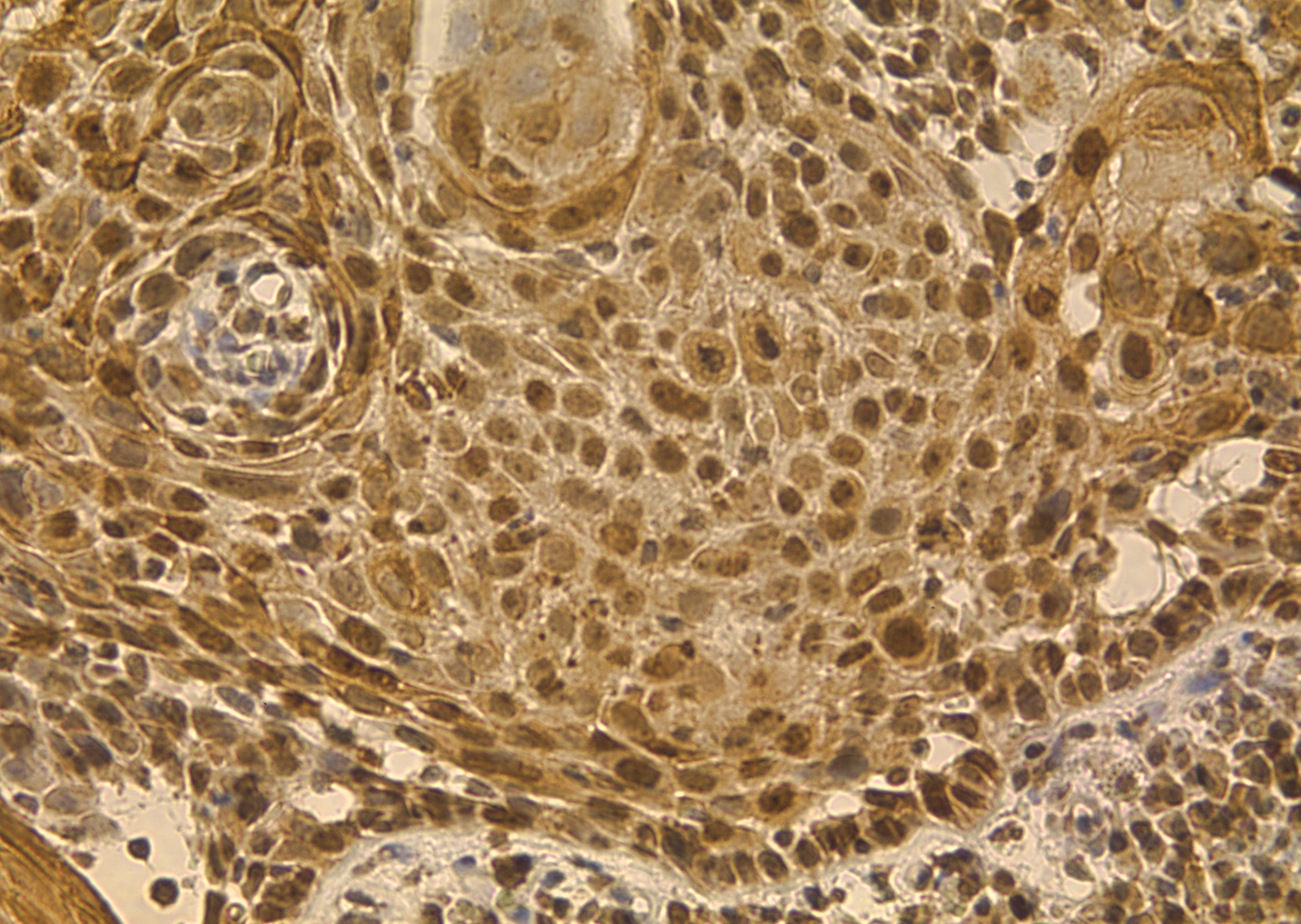

Supplement: S1 File — (ZIP) [file pone.0303120.s001.zip › Fig 7/Fig 7A 5 and Fig 7B 5.jpg]

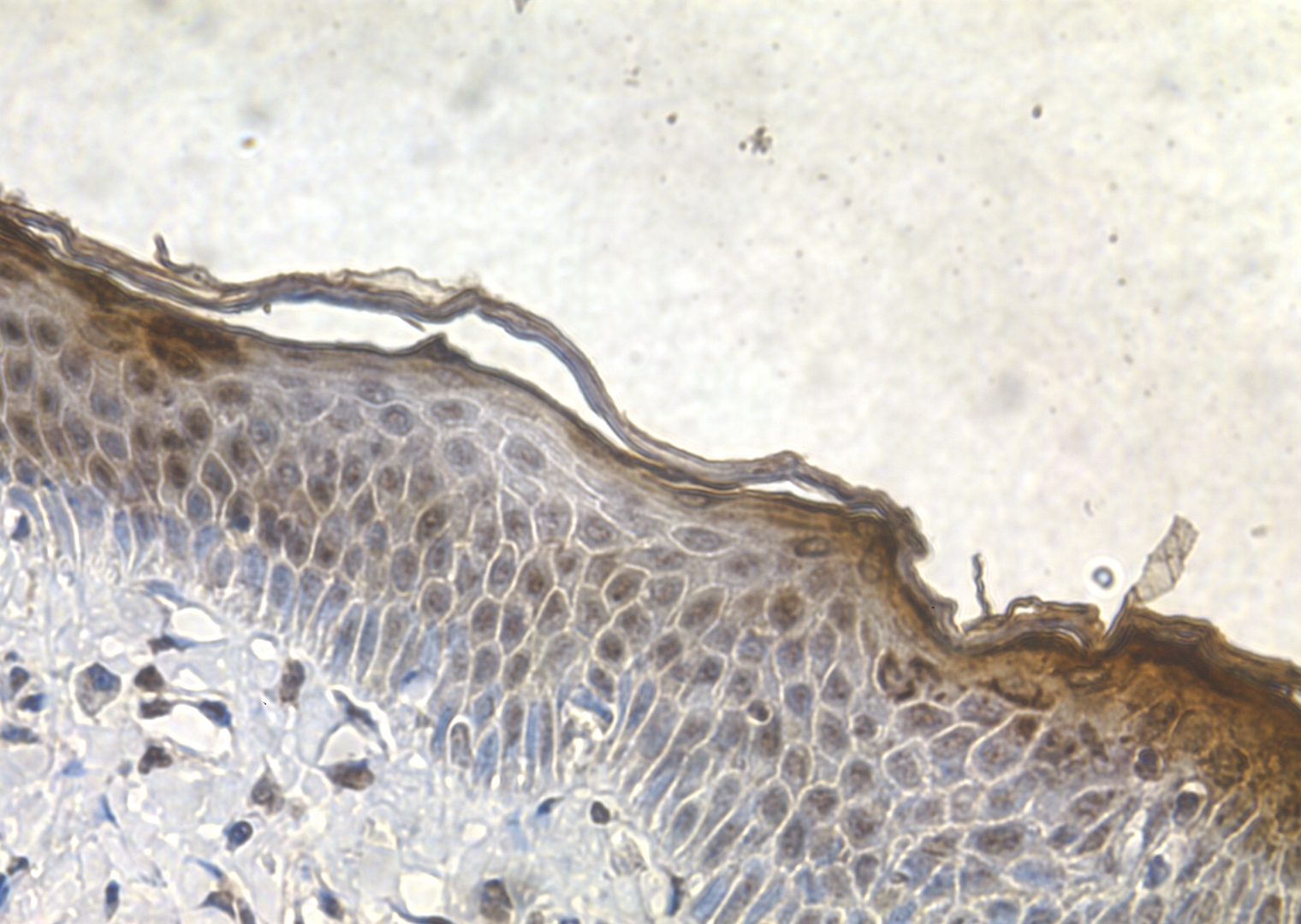

Supplement: S1 File — (ZIP) [file pone.0303120.s001.zip › Fig 7/Fig 7B 2.jpg]

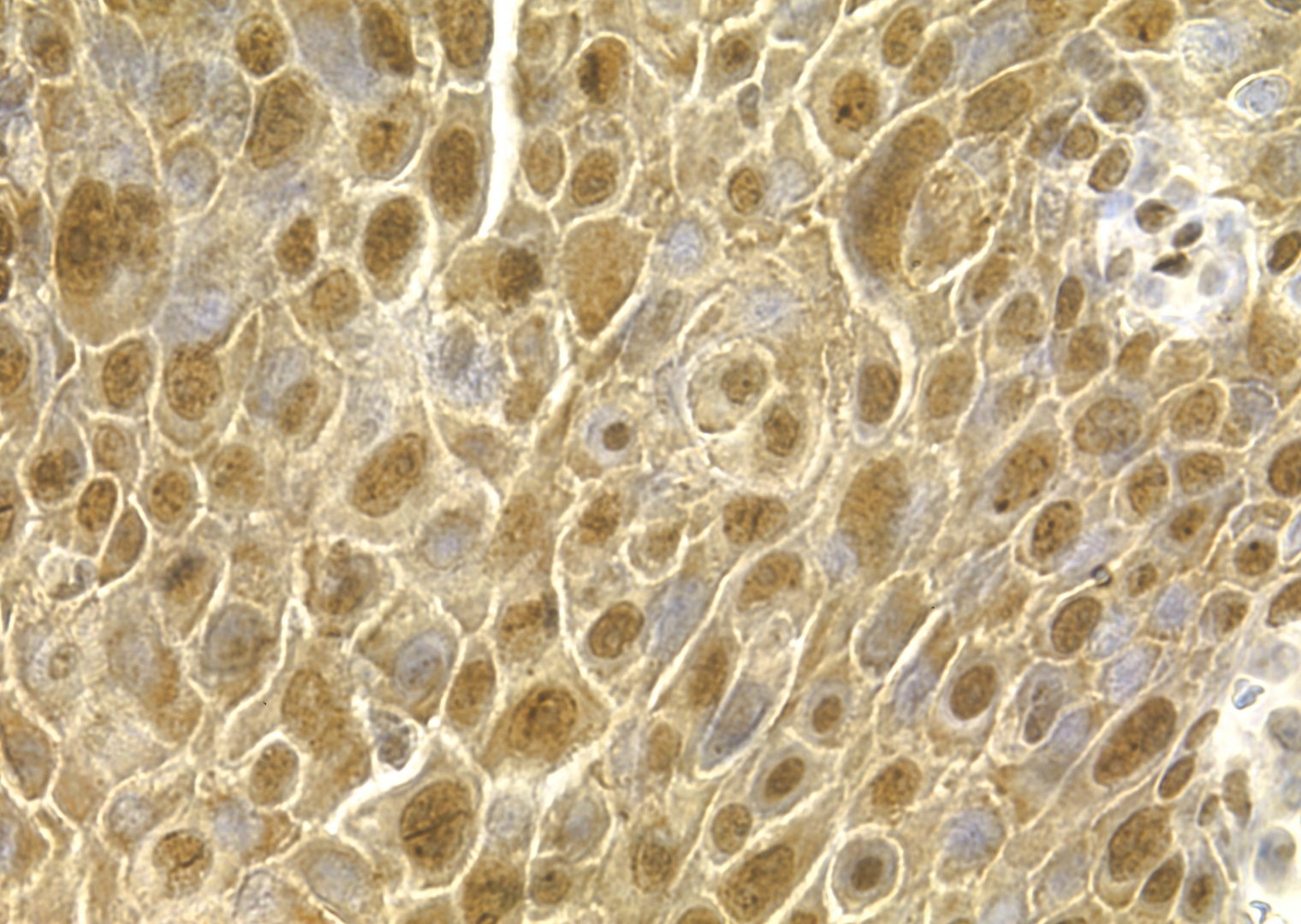

Supplement: S2 File — (ZIP) [file pone.0303120.s002.zip › Fig 9/Fig 9A lower.jpg]

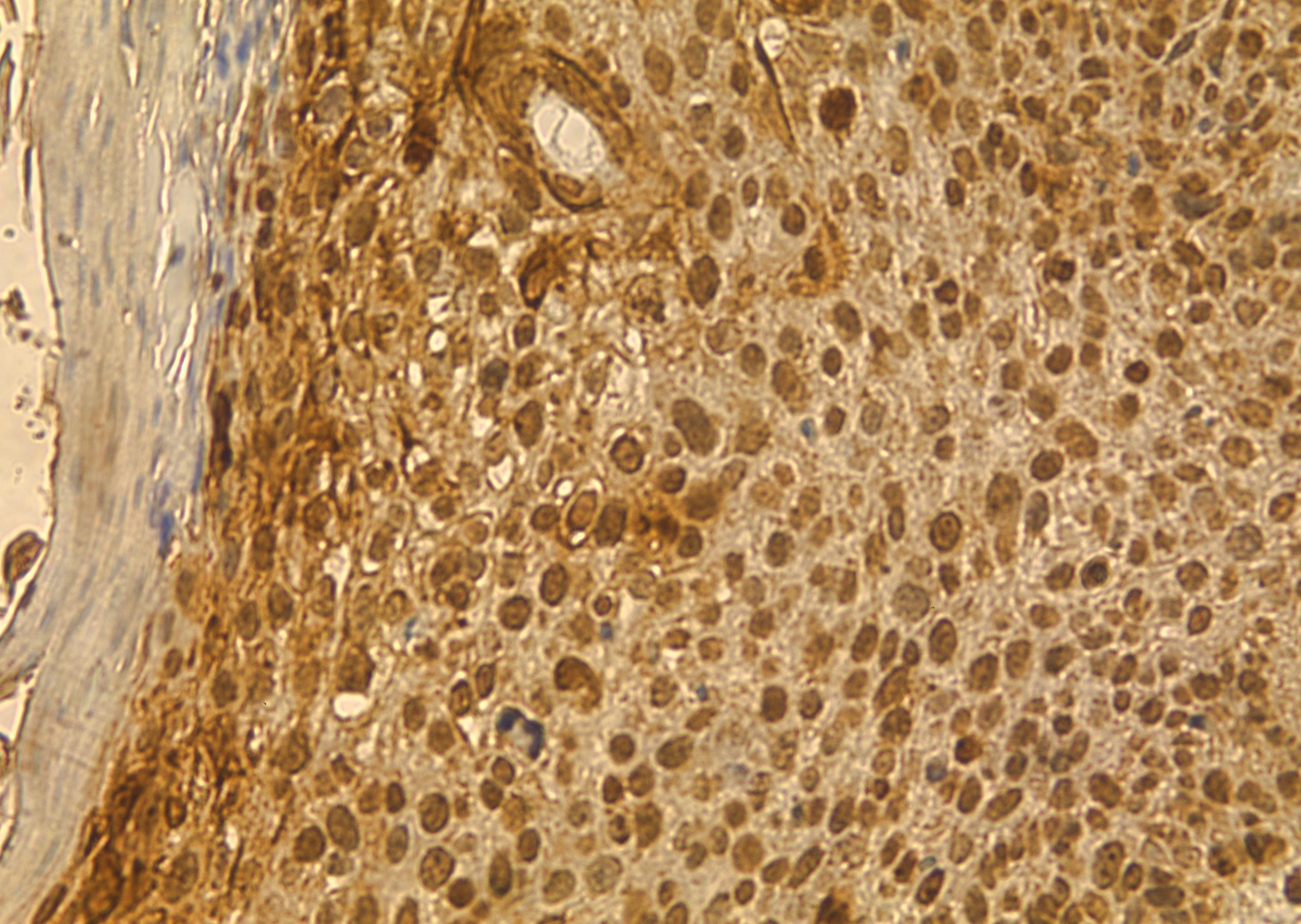

Supplement: S2 File — (ZIP) [file pone.0303120.s002.zip › Fig 9/Fig 9A upper.jpg]

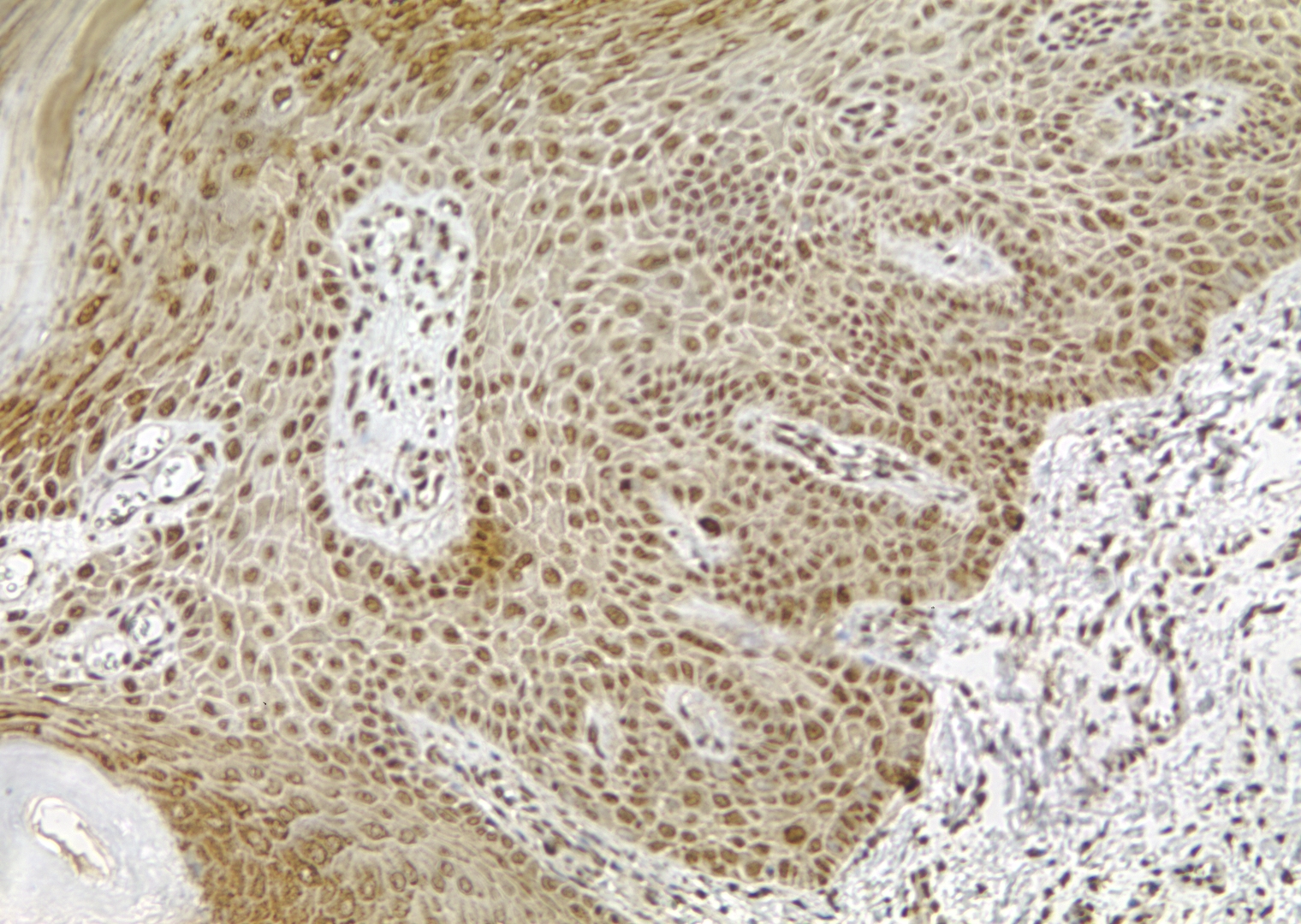

Supplement: S2 File — (ZIP) [file pone.0303120.s002.zip › Fig 9/Fig 9B upper.jpg]

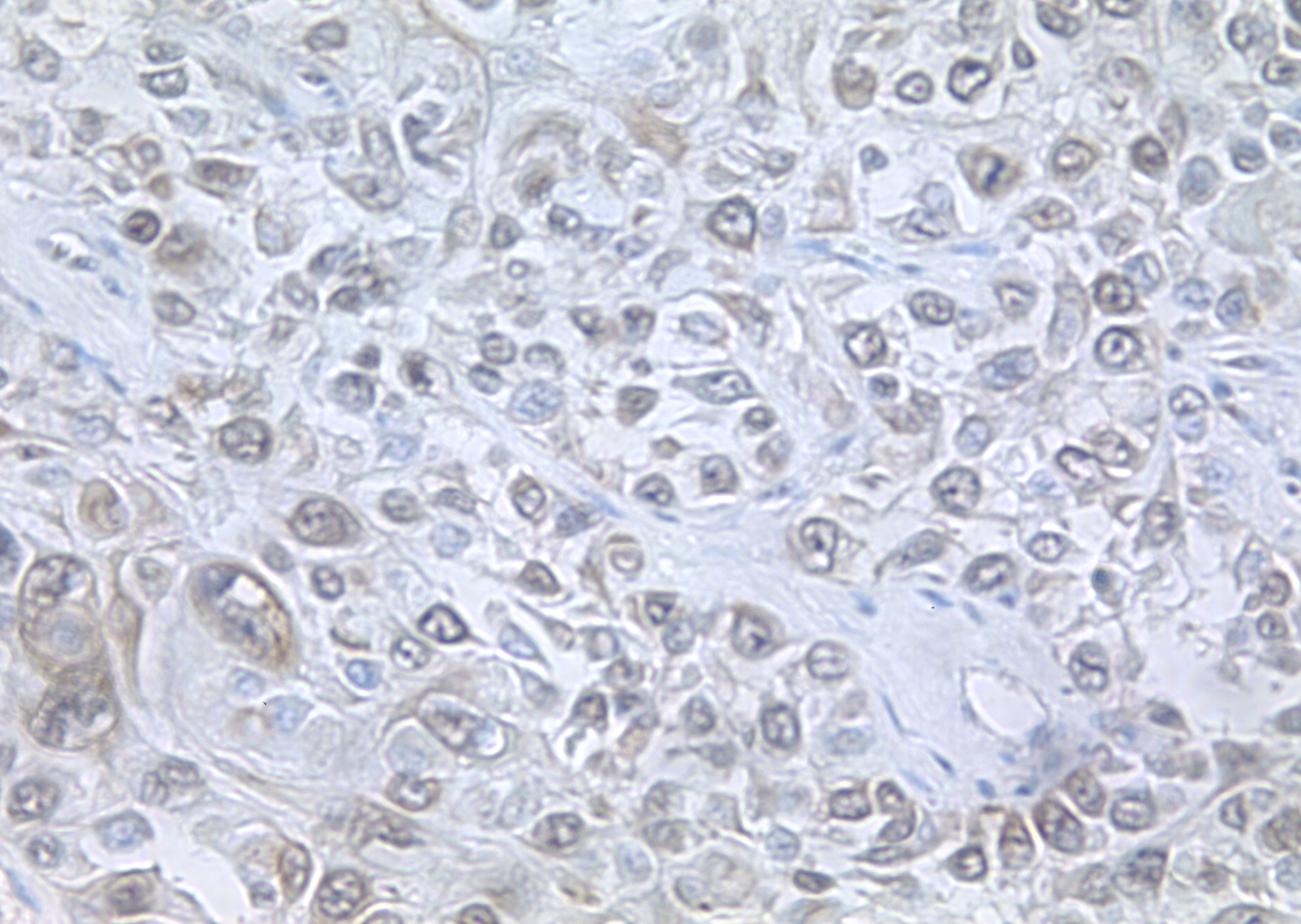

Supplement: S3 File — (ZIP) [file pone.0303120.s003.zip › Fig 10/Fig 10B lower.jpg]

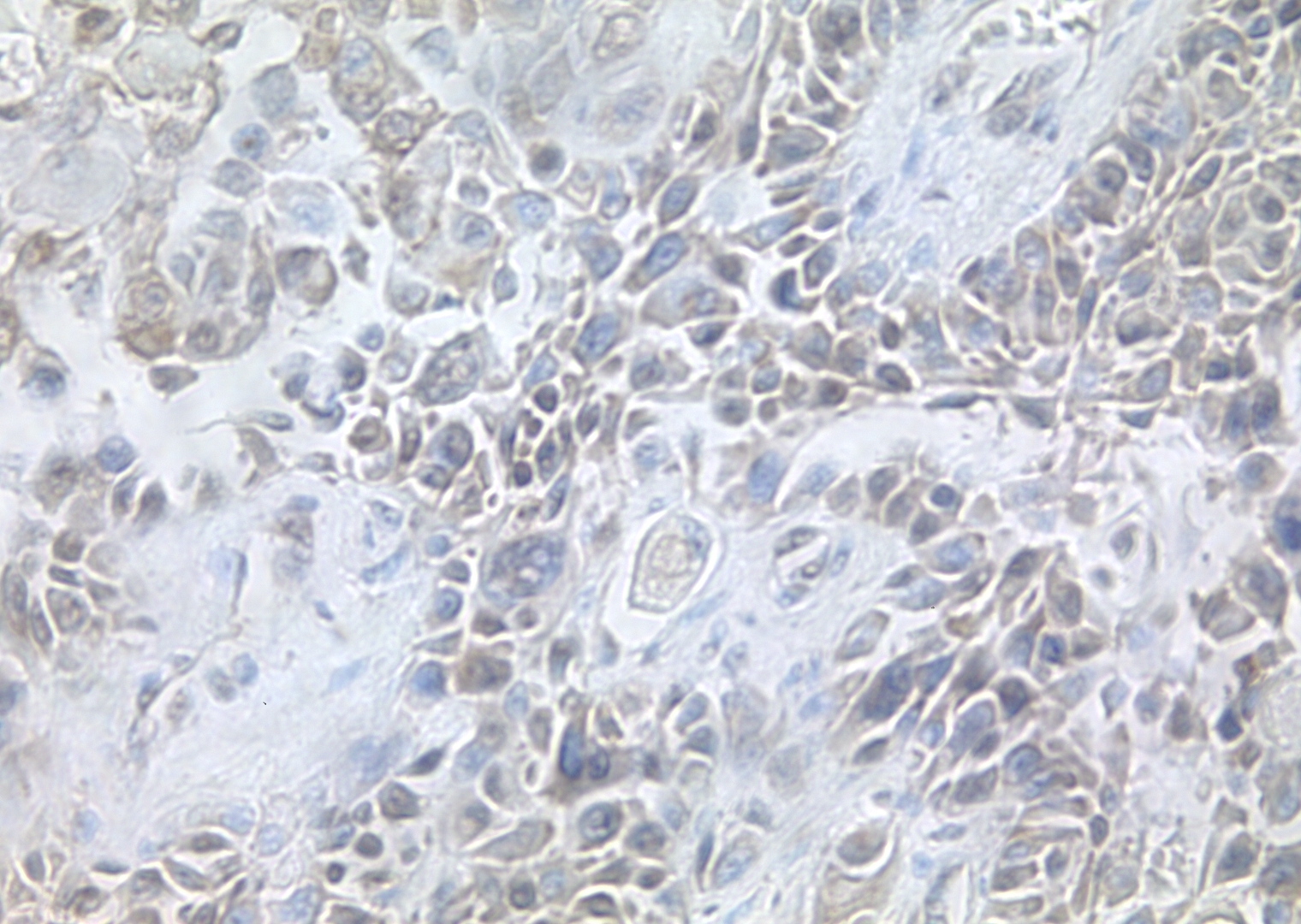

Supplement: S3 File — (ZIP) [file pone.0303120.s003.zip › Fig 10/Fig 10B upper.jpg]

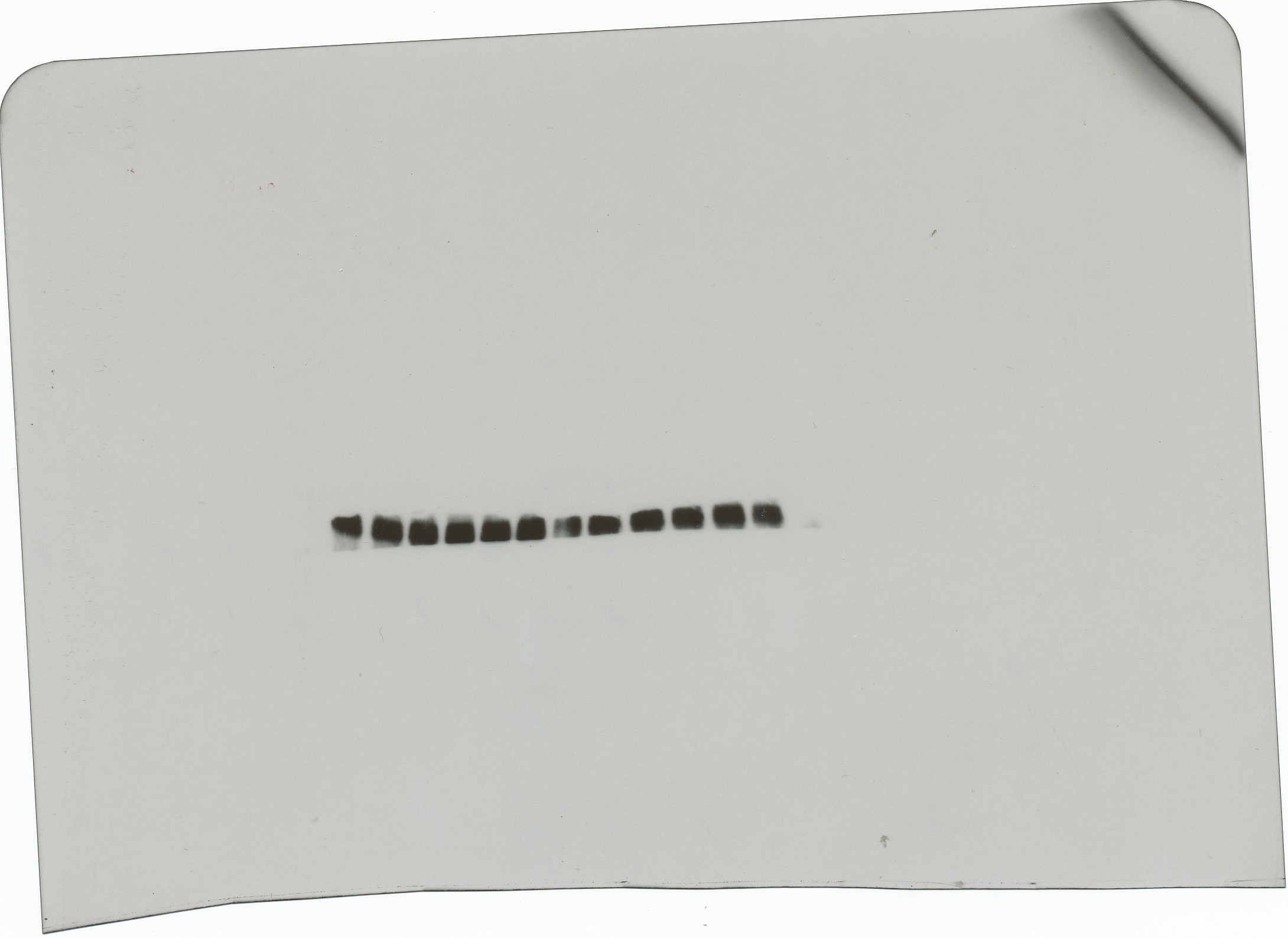

Supplement: S4 File — (ZIP) [file pone.0303120.s004.zip › S4/Bactin_loading_control.jpg]

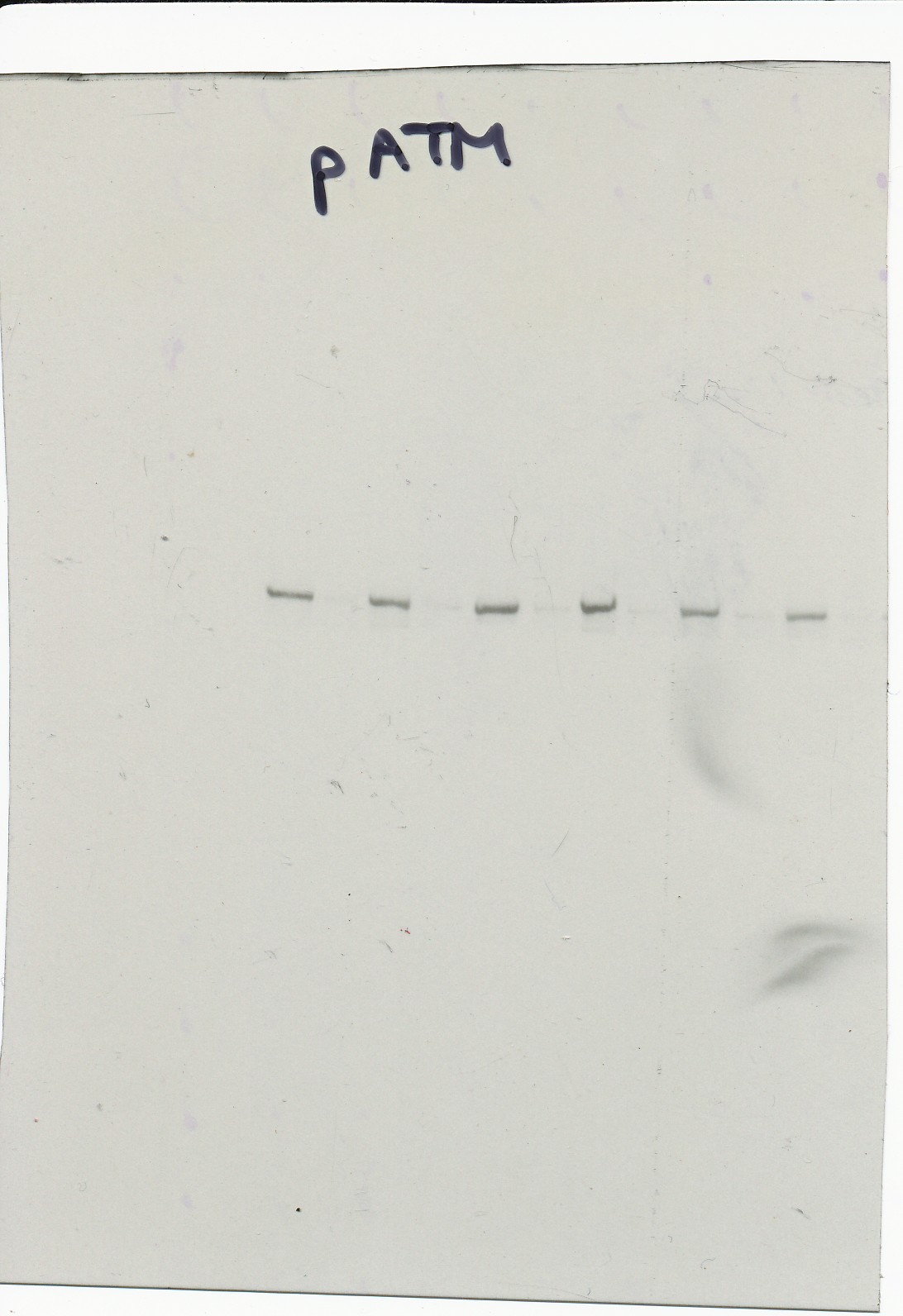

Supplement: S4 File — (ZIP) [file pone.0303120.s004.zip › S4/pATM_blot.jpg]
